# Nature prescribing: emerging insights about reconciliation-based and culturally inclusive approaches from a tricultural community health centre

**DOI:** 10.24095/hpcdp.44.6.05

**Published:** 2024-06

**Authors:** Anita Vaillancourt, Rebecca Barnstaple, Natalie Robitaille, Taylor Williams

**Affiliations:** 1 School of Social Work, Faculty of Health and Behavioural Sciences, Lakehead University, Orillia, Ontario, Canada; 2 Community Programs and Engagement, Chigamik Community Health Centre, Midland, Ontario, Canada; 3 School of Interdisciplinary Science, McMaster University, Hamilton, Ontario, Canada; 4 School of Nursing Nipissing University, North Bay, Ontario, Canada

**Keywords:** nature prescribing, nature connection, culturally inclusive, decolonization

## Abstract

This commentary highlights the importance of social and nature prescribing programs reflecting culturally diverse perspectives and practices. Creating and holding space for Indigenous and other worldviews should be a key priority of nature prescribing, a relatively recent practice in Canada that recognizes and promotes health benefits associated with engaging in a variety of activities in natural settings. Central to designing and delivering nature prescribing that is culturally inclusive and grounded in fulfilling obligations of reconciliation is recognizing the ongoing dominance of Western worldviews and their associated implications for decolonizing and Indigenizing nature-based programming. Consciously working to expand Western values, with the aim of extending nature prescribing practices beyond mere nature exposure to fostering emotional connections to nature, is a critically important part of the ongoing development of nature-based interventions and nature prescribing.

**Highlights**

- Nature prescribing is an increasingly recognized aspect of social prescribing that acknowledges and promotes enhanced health benefits associated with natural settings to address illness and promote health and wellness.
- The Western worldview maintains a narrow view of human relations with nature, consisting of humancentric needs and interests. The limited priority that the Western worldview places on the relationship with nature and the importance of establishing and maintaining nature connection, may limit the potential reach and benefits of nature prescribing.
- Nature prescribing efforts should recognize pre-existing, nature-based approaches such as land-based healing practised by Indigenous people and ensure culturally inclusive design and practices.

## Introduction

The benefits of nature exposure are widely recognized^1^ and include a range of positive physical and mental health outcomes such as reductions in stress responses, lowered blood pressure, reduced symptoms of anxiety and depression, and increases in physical activity such as walking.^2^,^3^ Nature prescribing is gaining momentum as a treatment modality for a range of chronic conditions including various mental health issues. Like social prescribing, nature prescription maintains similar objectives, such as reducing chronic disease burdens^1^ and redirecting nonmedical issues away from the primary health care system by leveraging social care resources and supports to address nonmedical, nonclinical health.^1^

Nature prescriptions are generally provided by a health care or social service provider who recommends a specific period of time for the individual to spend in a natural setting.^4^ Following the lead of other nations such as the United Kingdom and, more recently, the United States in their creation of social and nature prescribing programs, Canada has very recently engaged in developing nature prescribing, with most provinces offering programs in various stages of development. Ontario established formal social prescribing initiatives as early as 2018, and nature prescribing in British Columbia commenced in 2020 with the “PaRx” initiative of the BC Parks Foundation, with other provinces following suit; for example, Quebec with Prescri-Nature in 2023. 

Despite the utility of drawing upon established practice and programming from other nations, factors specific to Canada must be recognized in order to appropriately respond to the diverse cultural issues and needs related to social and nature prescribing. Historical and contemporary colonialism that continues to impact and disadvantage Indigenous and other racialized groups manifests in ways that are unique to Canada. 

A key issue is the legacy of settler colonialism, a system of colonization in which the colonizers not only settle on the invaded territory permanently, but work to establish themselves as naturalized and the legitimate occupants of the land.^5^ This type of colonialism is unique to Turtle Island (North America) and has been deeply damaging to Indigenous Peoples by dispossessing them of their land base and, by extension, disrupting their cultural identities, traditions, languages and spiritual connection to their traditional territories.^5^ Assimilation strategies and the resulting dominance of Western worldviews and colonial logics continue to reinforce economic, political, social and health inequities and disadvantages experienced by Indigenous Peoples. These challenges are widely attributed to the disconnection of Indigenous people from their ancestral lands^6^ and the ongoing marginalization of Indigenous worldviews within the Western context.^7^,^8^ Acknowledging the historical and ongoing impacts of settler colonization is therefore critical to developing culturally relevant and appropriate nature prescribing programming and practices.

This commentary draws on insights gained from the social and nature prescribing program developed (and practised) at Chigamik Community Health Centre (CHC) alongside a research partnership with Lakehead University. In particular, this discussion identifies several important issues and considerations surrounding design and practice implications for Indigenous people and inclusive nature prescribing. As this program is informed by a unique local context that includes Indigenous participants, practitioners and partners, it has led to the consideration that while Western models of “nature” and “green” prescribing contain elements that may reflect some aspects of Indigenous practices, such as land-based healing, important distinctions remain in terms of terminology, purpose, scope, framing and intent.

Chigamik CHC is a tricultural organization that provides primary and allied health care to Indigenous, Francophone and other historically marginalized community members of North Simcoe Muskoka, Ontario. In the spring of 2023, Chigamik CHC implemented a novel social prescribing program aimed at supporting better mental health through client-centred co-design and strengthened community supports. Thus far, the focus of nature prescribing at Chigamik has been the facilitation of access to locally identified natural areas through reducing barriers such as membership costs and transportation. Concurrently, Chigamik has expanded land-based healing programs designed for and by First Nation and Mtis community members, enhancing capacity through an increase in opportunities, resources and dedicated support staff. The overlap in intent to support holistic health and well-being through client-centred program design has led to considering similarities and differences in these programs and worldviews, and the role a land-based or nature context plays in social prescribing. Situating nature prescribing programs to reflect diverse community voices, we propose that cultural conceptions of “nature” be considered and accounted for in both the language and the type of social prescription, the expected outcomes, and the mechanism of action. Beyond “green and blue [water]” prescribing, much of which has focussed on physical activity and stress reduction, being in nature or with the land also embodies the capacity to function relationally. This occurs as an aspect of identity, and as a profound locus for meaning making and support that may mirror or transcend Western concepts of social connection. These aspects may also foster other benefits such as reductions in isolation and loneliness, elements that are notably the primary outcome measures for Chigamik CHC’s current initiative, which aims to improve mental health through social prescribing. Connectedness to nature has been shown to promote well-being and pro-environmental behaviours that can foster engagement and responsible relations with nature.^9^

## What is nature prescribing?

Nature prescribing is emerging as a significant aspect of social prescribing, with a range of terms and concepts associated with these practices. However, there is not a universal definition,^1^ with the result that nature prescribing is often used interchangeably with other terms, such as “green prescribing,” “green social prescribing” and “nature-based social prescribing,” and described as time spent in green spaces such as parks, grasslands, forests or gardens.^10^ Stanhope and Weinstein^11^ point out that the lack of specificity and conflation of green prescriptions with nature-based prescribing has led to confusion surrounding meaning, and mistaken attribution of study results in which lifestyle changes such as increased physical activity have been used to support the effectiveness of nature-based activities. In their systematic analysis of human health benefits associated with forest activities, Park et al.^12^ identified four types of activities: staying, walking, exercise and indirect exposure. They also specified that forest-based interventions differ from “mere experiences,” as they are intentionally designed by experts to achieve direct health benefits. 

Much of the research reported on nature prescribing tends to focus on physical activity and stress reduction occurring in a natural setting, rather than relational aspects or meaning making, reducing nature to a setting for activities that could happen elsewhere, while suggesting that effects may be enhanced by their occurring in an outdoor environment. In their narrative review, Jiminez et al.^13^ suggest that potential pathways through which nature may influence health include increased opportunities for social engagement and space for physical activity, removed from harmful effects of air pollution, noise and heat. 

Note that the emphasis here is on social engagement between people and does not extend to relations with nature itself. The limited focus on nature connection or relationality reflects constraints within the dominant Western paradigm that may limit the effectiveness of interventions for diverse populations. Congruence of the worldviews underlying nature prescribing program goals, objectives and practices with those of program participants is important to ensure inclusion, but also to ensure respect for other worldviews and as a means to inform and expand Western conceptualizations about nature and associated stewardship responsibilities. 

## Land-based healing

In contrast with the Western conceptions of nature and the practice of nature or green prescribing, which focusses on holding wellness activities in natural spaces, with little to no attention to relationality to nature or nature connection, “land-based healing” is widely practised in Indigenous communities. Land-based healing combines Indigenous knowledge and cultural traditions to help people heal. Land is crucial for cultural preservation and as a place of self-expression and traditional survival. Accordingly, land-based healing programs have become effective therapies for mental health, addiction treatment and complex trauma recovery by reconnecting Indigenous people with their ancestral lands, identities and traditions.^14^ In recognition of the impacts of colonization on Indigenous people, steps are taken in land-based healing to identify how an individual’s or a community’s relationship with the land, self and others has been disrupted and how best to help renew this relationship.

In contrast with green prescriptions that appear to frame nature as an objective site or location for a physical activity that is considered to be the active factor in supporting health, land-based healing takes place on intentionally spiritually cultivated, honoured and respected land.^14^ Land-based practices are defined as the profound interconnection between Indigenous epistemology and pedagogy, where the land assumes a pivotal role.^14^

For Indigenous peoples, aspects of the land are seen as fundamental parts of their identity and health.^15^-^19^ The land has a multitude of meanings that incorporate the interconnected physical, symbolic, spiritual and social aspects of their cultures.^19^,^20^ This concept surrounds all elements of the natural realm, encompassing plants, animals, ancestors and spirits, as well as various environmental components such as air, water, earth and minerals.^21^ Fostering a reconnection with ancestral territories holds significant relevance in advancing the promotion and intervention efforts aimed at enhancing the mental well-being of Indigenous populations.^22^,^23^ Intrinsic to land-based healing and all relations Indigenous Peoples have with the land is the principle of relational accountability, which acknowledges human beings as part of nature, interdependent with it rather than existing outside of it, with the responsibility to care for all aspects of nature to which we are related.^24^

## What is needed to ensure culturally inclusive, responsive and appropriate nature prescribing?

As a tricultural organization serving community members with diverse and often intersecting identities, Chigamik CHC aims to adopt and implement a Two-Eyed seeing approach,^25^ building on the strengths and perspectives of Indigenous and Western world views. Specific to growing acknowledgement by medical professionals that healing can be facilitated and enhanced through engagement with nature or land, it is imperative that we develop adequate and appropriate terms for what this means from a cultural perspective. Because relationships with nature can take many forms, both named and unspoken, space for discussions that promote nature connections to occur on an individualized basis is required. The ideal form for someone may be related to cultural identity, but it cannot be presumed that this is the sole determining factor.

Fostering a decolonized and Indigenized health equity approach to social and nature prescribing requires an ongoing awareness of the factors that contribute to inequities, including cultural determinants of health that influence engagement in meaningful activities. These factors also impact the likelihood of someone following a social or nature prescription or undertaking a change in behaviour. If the activity proposed is not within an appropriate framework, it is less appealing relevant and will potentially have less impact as a prescription or recommendation. Ensuring nature prescribing is reflective of worldviews is also critical to ensuring congruence between values, intentions and behaviours, which can further play a key role in influencing successful intervention outcomes.^26^

In addition, a commitment to reconciliation as demonstrated through concrete actions toward decolonizing nature prescribing in health care and addressing social determinants of health inequities should be a priority for Canadian organizations. This includes acknowledging factors that can constrain or facilitate access to nature. For example, Chigamik’s partner organization in social prescribing, Wye Marsh Wildlife Centre, recently announced free trail access for First Nations, Mtis and Inuit people to promote land access.

## Conclusion

The rapidly growing field of social and nature prescribing in Canada requires attention to diverse cultural perspectives as well as a firm commitment to health equity, social justice and reconciliation to ensure program design and practices reflect diverse local perspectives and needs. Recognizing historical and contemporary colonial relations and incorporating decolonizing and Indigenizing strategies within the terminology, program design, implementation and evaluation are also paramount to ensuring nature prescribing practices foster health and wellness benefits across Indigenous and non-Indigenous populations.

## Acknowledgements

No funding sources were received in support of preparing this commentary.

## Conflicts of interest

None of the authors have any conflicts of interest.

## Authors’ contributions and statement

AV, RB—conceptualization.

AV, RB—data curation.

AV, RB (equal)—methodology.

AV, RB—project administration. 

AV, RB (equal)—resources.

AV (project overall), RB (direct student supervision)—supervision.

AV, RB (equal)—validation.

AV, RB—visualization.

AV, RB, & NR, TW (equal)—writing—original draft.

AV, RB, & NR, TW (equal)—writing—review and editing.

The content and views expressed in this article are those of the authors and do not necessarily reflect those of the Government of Canada.
